# Supplementary material for: The association between Dioscorea sansibarensis and Orrella dioscoreae as a model for hereditary leaf symbiosis
Source: PLoS One. 2024 Apr 22;19(4):e0302377. doi: 10.1371/journal.pone.0302377 (PMC11034651; doi:10.1371/journal.pone.0302377)
Supplement: S1 Fig — (A) Node cuttings were taken from adult plants and incubated for 8 hours in 5% PPM for initial sterilization. (B) Node cuttings were incubated in a mixture of liquid MS, antibiotics and PPM for 3 weeks. (C) After 3–4 weeks, a bulbil (b) with its root system became apparent. Multiple leaves have formed from the node and are providing sugars to the plant. (D) The bulbil grows its own stem (s) that uses gravitropism to grow up and after the emergence of 2 leaves, the apical bud becomes visible. (E) After confirmation of being aposymbiotic by crushing and plating out the newly developed acumen(s), the plant was re-inoculated with a bacterium of interest by dropping 2 μl of the bacterial suspension on the apical bud. (PDF) [file pone.0302377.s001.pdf]

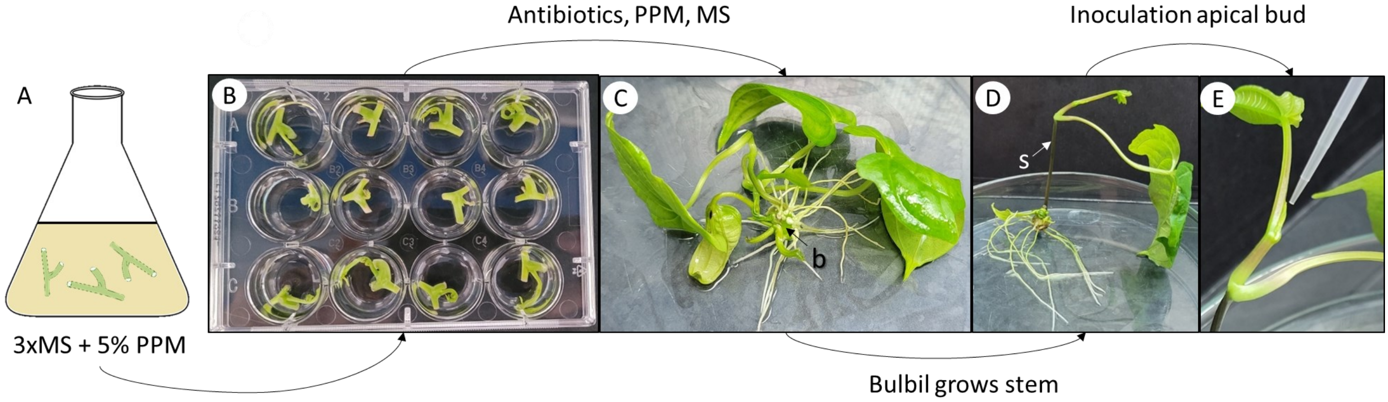

Figure S1: Method developed to make aposymbiotic plants and re-introduce a bacterium of interest. (A) Node cuttings were taken from adult plants and incubated for 8 hours in 5% PPM for initial sterilization. **(B)** Node cuttings were incubated in a mixture of liquid MS, antibiotics and PPM for 3 weeks. **(C)** After 3-4 weeks, a bulbil (b) with its root system became apparent. Multiple leaves have formed from the node and are providing sugars to the plant. **(D)** The bulbil grows its own stem (s) that uses gravitropism to grow up and after the emergence of two leaves, the apical bud becomes visible. **(E)** After confirmation of being aposymbiotic by crushing and plating out the newly developed acumen, the plant was re-inoculated with a bacterium of interest by dropping 2 µl of the bacterial suspension on the apical bud.
