# Supplementary material for: The association between Dioscorea sansibarensis and Orrella dioscoreae as a model for hereditary leaf symbiosis
Source: PLoS One. 2024 Apr 22;19(4):e0302377. doi: 10.1371/journal.pone.0302377 (PMC11034651; doi:10.1371/journal.pone.0302377)
Supplement: S2 Fig — Wild-type colonized D. sansibarensis were inoculated by a O. dioscoreae R-71412 cell suspension (Orrella) or a sterile 0.4% NaCl solution (MOCK) and grown for 4 weeks in gnotobiotic conditions. Leaf surface area (A) and length of the forerunner tip containing the bacterial glands (B) were measured for 3 leaves per plant, starting with the leaf closest to the shoot tip (leaf 1, not shown). C. Total stem length measured from the crown to the shoot tip. Data from 2 independent experiments are shown separately. Data from mock-inoculated plants are shown in orange, and in blue for O. dioscoreae-inoculated plants. The distributions of values between the O. dioscoreae–or mock-inoculated plants are identical for each of the 3 parameters (Wilcoxon rank sum test p > 0.05). (PDF) [file pone.0302377.s002.pdf]

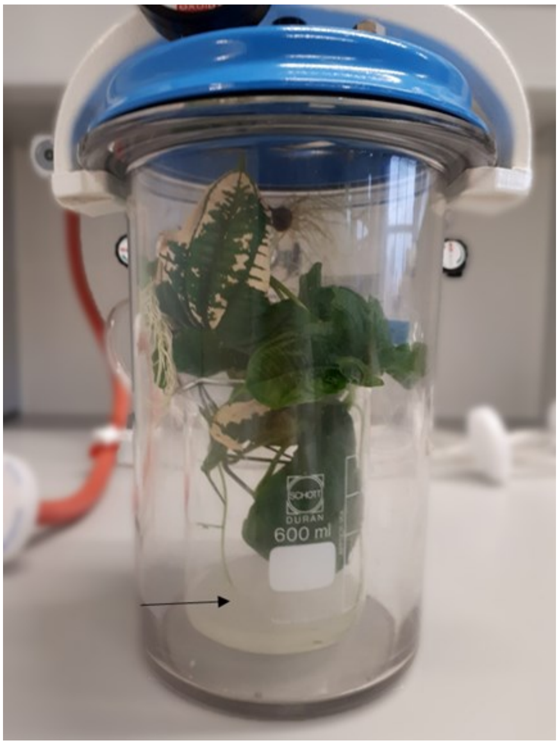

Figure S2: inoculation of *D. sansibarensis* in a vacuum chamber.
Wild-type colonized *D. sansibarensis* was inoculated by a liquid R-71416 culture by dipping the shoot tip (arrow) in the liquid culture while lowering the atmosphere pressure to 0.53 bar using a vacuum chamber.
