# Supplementary material for: The association between Dioscorea sansibarensis and Orrella dioscoreae as a model for hereditary leaf symbiosis
Source: PLoS One. 2024 Apr 22;19(4):e0302377. doi: 10.1371/journal.pone.0302377 (PMC11034651; doi:10.1371/journal.pone.0302377)
Supplement: S4 Fig — Wild-type colonized D. sansibarensis were inoculated by a O. dioscoreae R-71412 cell suspension (Orrella) or a sterile 0.4% NaCl solution (MOCK). Physiological parameters were measured using a hand-held optical meter after 4 weeks of growth in gnotobiotic conditions. Parameters measured include A. Chlorophyl content (Chl); B. Anthocyanins index, measured as a function of green light absorbed by the sample; C. Flavonoids index (Flav), measured as a function of UV light absorbed by the sample and D. Nitrogen Balance Index (NBI) is measured as the ratio of Chl and Flav and is an indicator of C/N allocation changes due to N-deficiency. Data from 2 independent experiments are shown separately. Data from mock-inoculated plants are shown in orange, and in blue for O. dioscoreae-inoculated plants. The distributions of values between the O. dioscoreae–or mock-inoculated plants are identical for each of the 4 parameters (Wilcoxon rank sum test p > 0.05). (PDF) [file pone.0302377.s004.pdf]

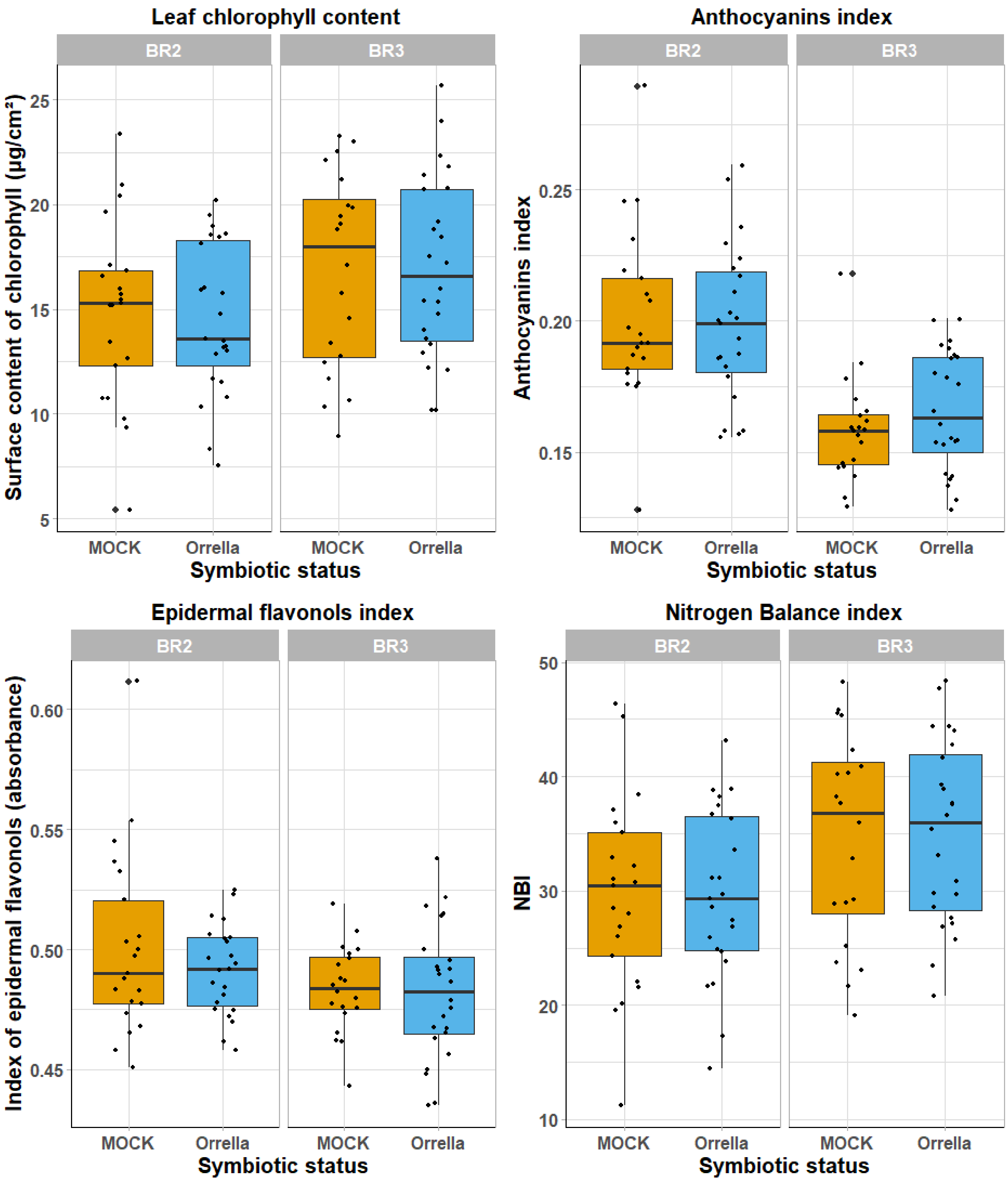

Figure S4: Physiological parameters of aposymbiotic vs. symbiotic *D. sansibarensis* in gnotobiotic conditions. Wild-type colonized *D. sansibarensis* were inoculated by a *O. dioscoreae* R-71412 cell suspension (Orrella) or a sterile 0.4% NaCl solution (MOCK). Physiological parameters were measured using a hand-held optical meter after 4 weeks of growth in gnotobiotic conditions. Parameters measured include **A**. Chlorophyll content (Chl); **B**. Anthocyanins index, measured as a function of green light absorbed by the sample; **C**. Flavonoids index (Flav), measured as a function of UV light absorbed by the sample and **D**. Nitrogen Balance Index (NBI) is measured as the ratio of Chl and Flav and is an indicator of C/N allocation changes due to N-deficiency. Data from 2 independent experiments are shown separately. Data from mock-inoculated plants are shown in orange, and in blue for *O. dioscoreae*-inoculated plants. The distributions of values between the *O. dioscoreae*– or mock-inoculated plants are identical for each of the 4 parameters (Wilcoxon rank sum test $p > 0.05$).
