# Supplementary material for: The association between Dioscorea sansibarensis and Orrella dioscoreae as a model for hereditary leaf symbiosis
Source: PLoS One. 2024 Apr 22;19(4):e0302377. doi: 10.1371/journal.pone.0302377 (PMC11034651; doi:10.1371/journal.pone.0302377)
Supplement: S1 Table — (PDF) [file pone.0302377.s005.pdf]

Table S1: Bacterial species used in this study

Species	Strain or plasmid	Description	Growth conditions	Reference or source
Orrella dioscoreae	R-71417	Strain R71412 with mini Tn7(Gm) Ptac-mCherry	TSA + Nalidixic acid 30 µg/ml + Gentamycin 20 µg/ml, 28°C,	[31]
Orrella dioscoreae	R-71416	Strain R71412 with mini Tn7(Gm) Ptac-GFP	TSA + Nalidixic acid 30 µg/ml + Gentamycin 20 µg/ml, 28°C,	[31]
Orrella dioscoreae	R-67584	Accession nr: 19760001, plant from direct wild origin, Origin: Congo DR	TSA, 28°C, aerobic	Evrard C., Louvain-la-Neuve, U.C.L., Ecologie et Biogeographie,
Orrella dioscoreae	R-67173	Isolated from leaf nodules of *Dioscorea sansibarensis* (Zanzibar yam)	TSA, 28°C, aerobic	[23]
Orrella dioscoreae	R-67088	isolate from leaf nodules of *Dioscorea sansibarensis,* botanical garden of the University of Ghent,	TSA, 28°C, aerobic	[23]
Orrella dioscoreae	R-67090	isolate from leaf nodules of *Dioscorea sansibarensis,* botanical garden of the University of Ghent,	TSA, 28°C, aerobic	[23]
Orrella dioscoreae	LMG-29303^{T}	Type strain *O. dioscoreae*	TSA, 28°C, aerobic	[23]
