# Supplementary material for: The association between Dioscorea sansibarensis and Orrella dioscoreae as a model for hereditary leaf symbiosis
Source: PLoS One. 2024 Apr 22;19(4):e0302377. doi: 10.1371/journal.pone.0302377 (PMC11034651; doi:10.1371/journal.pone.0302377)
Supplement: S2 Table — (PDF) [file pone.0302377.s006.pdf]

Table S2: Minimum inhibitory concentrations of biocidal products on different *O. dioscoreae* strains

Concentrations tested :

	**Carbenicillin**	**Cefotaxime**	**PPM**
**R-67173**	64	128	0,04
**R-67584**	64	128	0,04
**R-67088**	32	128	0,04
**R-67090**	32	128	0,04
**LMG 29303^T**	32	128	0,04
