# Supplementary material for: The association between Dioscorea sansibarensis and Orrella dioscoreae as a model for hereditary leaf symbiosis
Source: PLoS One. 2024 Apr 22;19(4):e0302377. doi: 10.1371/journal.pone.0302377 (PMC11034651; doi:10.1371/journal.pone.0302377)
Supplement: S3 Table — APO = aposymbiotic status, SYM = symbiotic status, check-ups quantified the amount of O. dioscoreae found in new leaf acumens. Not = Majority isolates not identified as O. dioscoreae. Last column gives the eventual identity given to the sample for further analysis: APO = aposymbiotic plant, unknown = colonized by bacteria other than O. dioscoreae, Orrella dioscoreae = colonized by Orrella dioscoreae. (PDF) [file pone.0302377.s007.pdf]

Table S3: Symbiotic status of plants used in phenotyping experiment. APO= aposymbiotic status, SYM= symbiotic status, check-ups quantified the amount *of O. dioscoreae* found in new leaf acumens. Not= Majority isolates not identified as *O. dioscoreae.* Last column gives the eventual identity given to the sample for further analysis: APO= aposymbiotic plant, unknown= colonized by bacteria other than *O. dioscoreae, Orrella dioscoreae* = colonized by *Orrella dioscoreae*

Plant ID	Symbiotic status	inoculated with	Status T0	Status T1	Status T2	check-up 3B	Status 3 Identification	Final status
1	APO	Mock	APO	0	0	0		APO
2	APO	*O. dioscoreae*	APO	0	0	0		APO
4	SYM		SYM		0	0		Unknown
5	APO	Mock	APO	0	0	0		APO
6	APO	Mock	APO	0	0	0		APO
8	SYM				0	0		Unknown
9	APO	*O. dioscoreae*	APO	0	10^4	10^5	not	Unknown
10	SYM		APO		0	10^2	not	Unknown
11	APO	Mock	APO	0	0	1	not	APO
12	APO	*O. dioscoreae*	APO	0	10^3	10^3	*O. dioscoreae*	*O. dioscoreae*
13	APO	Mock	APO	0	0	10^2	not	APO
14	APO	*O. dioscoreae*	APO	0	0	10^2	*O. dioscoreae* + other	Unknown
15	APO	Mock	sym	0	10^3	10^3	not	Unknown
16	APO	*O. dioscoreae*	O. dioscoreae	10^3	10^3	10^3	*O. dioscoreae*	*O. dioscoreae*
17	APO	*O. dioscoreae*	*O. dioscoreae*	0	10^3	10^3	*O. dioscoreae*	*O. dioscoreae*
19	SYM		SYM		0	10^2	*O. dioscoreae*	*O. dioscoreae*
20	APO	Mock	SYM	0	0	10^1	*O. dioscoreae* + other	Unknown
21	SYM		SYM		0	10^2	*O. dioscoreae* + other	*Orrella dioscoreae*
22	APO	*O. dioscoreae*	indecisive	0	1	10^2	*O. dioscoreae* + other	*O. dioscoreae*
23	SYM		SYM		0	0		Unknown
24	SYM		SYM		0	0		Unknown
