# Supplementary material for: The association between Dioscorea sansibarensis and Orrella dioscoreae as a model for hereditary leaf symbiosis
Source: PLoS One. 2024 Apr 22;19(4):e0302377. doi: 10.1371/journal.pone.0302377 (PMC11034651; doi:10.1371/journal.pone.0302377)
Supplement: Attachment — Submitted filename: Review Accar 2023.pdf [file pone.0302377.s009.pdf]

Review Accar 2023

Main remarks

Really nice work, you can see that even though it's the most 'easy' interaction in the bacterial leaf symbiosis, it's still a handful to work with these symbiotic plants, especially due to their longer life cycle and difficult plants to propagate.

So congratulations for your manual work and results.

I have some remarks on the main text.

- There were a lot of beauty flaws in your work
  - Check your reference style
    - Some scientific names in the references are in italics, others are not.
  - Use your units consequently
    - if you use L, use than mL
    - Use spaces between amount and unit
    - Stick to which notation you want to use: 37°C, 37 °C, 20 g/L NaCl, 20g/l NaCl, NaCl 20 g/L or NaCl (20 g/L). All notations are well as long as you don't see them all in the same paper (R144 vs R145). Be consistent with the use of capitals and space.
  - Use sub and superscript when you write chemical formula (R184, R 244)
  - When are amounts written out in full? Under 10 (not followed by a unit) or at the beginning of a sentence? Both rules are sometimes followed. Be consistent
  - Be sure to explain your abbreviations in full before you use them.
    - MS(R108), TSB (R122), EMS (R173),…
    - Are some abbreviations really necessary? PO (R173) , RT (R180) or MIC (307)
- Are all the adjustments of your material & methods written in your results present in your material and methods? There is a strange flow during the material & methods, while the story comes clear in the results. I would advise to structure the m&m more, perhaps the use of a set the scene in the start of M&M could help the reader to show how you worked. In the methods all the methods seem to be done all at once, while the results show it was a process of elimination to be able to make a aposymbiotic plant.
- There is no data on the development of first part of the results (Symbiotic *D. Sansibarensis* are recalcitrant to inoculation with exogenously applied *O. dioscoreae*). Is it possible to add some data in the supporting information?
- It is not clear which statistical tests were used to validate the results. Can you specify and add these tests and the p-values?
- In the result section, references were added to support some methods, these references fit more in the method section than the result section.
- In the discussion, it is not clear which method was the best option to develop an aposymbiotic plant. In addition, none of the methods is sound, there is always a possibility of infection. Do you have ideas to further investigate and search for a better method?

1. Introduction

- R38 Be consistent. If you refer every time to a plant – bacteria interaction. It is maybe best to address the symbiosis as *Medicago/Sinorhizobium.* In the rest of your paper, you are talking of *D.sansibarennsis/ O. dioscoreae.*
- R69. Why is it important to state that nitrogen fixation is ruled out in the *Dioscorea*/*Orella* symbiosis, is it not in the other leaf nodulating interactions?

2. Material & Methods

- R108: ½ MS vs R115 half strength Murashige and Skoog
- R116 16h/8h routine, maybe use this abbreviation for the rest of the methods (R132, R151, R244)
- R117: The injection in the bulbils was it at a specific location and depth or random?
- R124-R130: Strange construction, wouldn't it be better to put the names of the different methods between brackets after each sentence?
- R134 Could it be possible to state that you will use two surface sterilisation protocols? The bleach+ ethanol protocol and the PPM protocol
- R143: you write what is present in MS medium, is that the same in R108, R115, R140?
- R143 2% sucrose is that (w/v) like R215?
- R153-R159: suggestion: would a table with all the *Orella* strains, with the specific characteristics not be a good addition?
- R153-159: why did you use both tags GFP en mCherry?
- R196 a verb is missing. Is this the triple A-staining mentioned in Figure 1?
- R236 'Leaf length, width, area, and acumen length were determined from photographs…' was this used to calculate the leaf area
- R240: which statistical analysis was used?
- R269: which statistical analysis were used?

3. Results

- If you are not combining results and discussion, it's strange to see references in this part. (R275) Could these references be integrated in the discussion or the methods??
- Table 1: Maybe add in the caption which effect you were researching and the contact time.
- R273-296: why were the amounts of bulbils and shoot tips not mentioned in the material and methods.
- R298-R328. This is a mix of Methods (some of the methods explained here are not mentioned in methods), results and discussion.
- R341 how did you test the presence of bacteria? Molecularly?
- Table 1:
  - maybe add contact time and which effect you were researching in your caption
  - 10°, does that mean that 1 or none CFU were found?
- Figure 1:
  - why no white circle around G en H?
  - why is the resolution different between G-H, I-J, M-N?
  - Caption:
    - what is triple A staining, not mentioned in the text

        - C and D, the mucus, trichomes and bacteria can they be visualised?
        - K and L, can the trichomes be visualised.
        - L remove (L)
        - M I would not abbreviate ER
- Figure 2:
    - Caption.
        - Is it not possible to emphasise more that the gland is recolonised?
        - *mcherry?* Is it necessary to put it in italics here cf. there results of the GFP-strain R277
- R392-R407: you mention 24 plants, while in the methods, you start with 25 plants. In addition you mention in the methods that 18 plants were aposymbiotic, while in the results you only mention 14.
- R398 which method of inoculation could give a better option, this dripping option was also not a good option with the aposymbiotic bulbils?
- R404 which statistical test did you use to test whether the results were significant.
- R405-R407: why do you refer first to B and C and then to A.
- Figure 3:
    - Caption refers to stem lengths in C, while the graph refers to shoot length
    - Caption refers to the total leaf area of individual plants tracked over a period of 30 days. What are the dots and what is the line?
    - C I would remove the abbreviations apo, control and sym if you work with a colour legend
    - C are the dots around the boxplots the values? Why are these not on one line per phenotype? These dots are not identified in the caption or in the legend.

4. Discussion

- R439 Not clear here, if leaf nodules in *Psychotria* develop when the bacteria is absent.
- R450 *Psychotria kirkii* is a synonym of *P. punctata*

5. References

6. Supporting information

- Figure S1:
    - Add white circles to make the letters readable and consistent.
    - S should be a small letter and the same font size as b. (maybe use arrows to visualise specific structures
    - I can't find s on the figure E
- Figure S2:
    - Is it possible to use a bigger font size on the axis
    - Why are the values spread over the X-axis, should be one line per condition (same remark as figure 3)
- Figure S3
    - Is it possible to use a bigger font on the axes
    - Use the same layout as Figure S2
    - Why are the values spread over the X-axis, should be one line per condition (same remark as figure 3)
- Table S1:

    - why not mentioned in the methods (R152-159)
    - Maybe add in which experiment they were used?
    - Why was *E.coli* used?
- The data underlying the statistics are missing, can these be added as well?
