# Supplementary material for: The association between Dioscorea sansibarensis and Orrella dioscoreae as a model for hereditary leaf symbiosis
Source: PLoS One. 2024 Apr 22;19(4):e0302377. doi: 10.1371/journal.pone.0302377 (PMC11034651; doi:10.1371/journal.pone.0302377)
Supplement: Attachment — Submitted filename: Response to reviewers.docx [file pone.0302377.s010.docx]

# Response to reviewers

### Editorial office:

Thank you for your suggestion to deposit our protocols to protocols.io. This is a very good idea and we will consider it.

1. We carefully read the PLOS ONE formatting guidelines and made sure that our revised manuscript met the requirements.

2. All relevant data is available in the manuscript or deposited in a public repository (see the Data availability statement)

3. We corrected the acknowledgment section as suggested.

Please find our amended Funding Statement below:

“This work was supported by the UGent Special Research Fund under grant BOFSTA2017002001 to AC. AC also acknowledges support from the French National Research Agency under grant agreement ANR-19-TERC-0004-01 and from the French Laboratory of Excellence project  "TULIP" (ANR-10-LABX-41; ANR-11-IDEX-0002-02). The TRI-FRAIB imaging platform facilities, FR AIB 3450 CNRS-UTIII, member of the national infrastructure France-BioImaging, are supported by the French National Research Agency (ANR[1] 10-INBS-04). The funders had no role in study design, data collection and analysis, decision to publish, or preparation of the manuscript.”

4. We are submitting our manuscript as an MS Word document, not a LaTeX file.

5. We apologize for the (sometimes unwarranted) use of the phrase “data not shown”. We amended the text to either remove these statements, or directly give the information to the reader.

6. Figure 1 and S1 include for illustrative purposes parts of images used to generate figures for an Acar et al. 2022 manuscript in the journal mBio, published under the Creative Commons CC BY 4.0 license. The manuscript authors (including the lead and corresponding authors of this manuscript) retained copyright of the images and allow unrestricted reuse of the material. We included the required attribution in the figure legend.

## Reviewer 1

(response from authors in highlighted font)

General Comments: This study deals with understanding the leaf symbiotic relationship between the host, Dioscorea sansibsarensis, and its bacterium, Orrella dioscoreae. This symbiotic relationship is unique among other symbiotic leaf taxa in other plant families as the study shows the bacterium can be cultured outside the plant, the plant can grow normally without the bacterium, and the same bacterium or genetically modified bacterium can be reintroduced into the uninfected plants. This experimental regime satisfies Koch’s postulates (which should be mentioned in the text). This experimental system may allow for a better understanding of other similar or obligate leaf symbioses as to their functional value(s).
Specific Comments: there are comments made throughout the attached, edited text. The monocot Dioscorea sansibsarensis symbiotic relationship seems significantly different from the obligate dicot leaf symbioses in that the bacteria are always on the leaf surface covered by the curled leaf lamina tip and associated secretory trichomes, and never encapsulated internally like with the nodules of Psychotria, Pavetta, and Ardisia sps. The enclosed shoot apices of the latter sps. do serve as an ‘external’ reservoir with stipular secretory glands/trichomes that maintain a bacterial population until it penetrates into the leaf lamina to form encapsulated nodules. However, none of these relationships show the bacteria inside cells but remain intercellular within the isolated nodules, a completely different environment than in this study. Whether further research on the system in the present study will shed light on the former obligate taxa remains an open question. With this said, the present study is worthy of publication because future research may uncover information that is relevant to it and the obligate taxa mentioned.
Title: appropriate;
Abstract: Identifies the purpose of the study and its rationale;
Key words: no key words listed in body but shown previously;
Introduction: Provides an excellent background for why the study is being conducted. It covers the key references and points out its significance related to previous studies;
Material and methods: See various comments. the M&m are presented in great detail by individual sections, and can be readily reproduced by other researchers, if necessary;
Results: See comments throughout Results;
Discussion: covers the Results adequately. It would be useful to include adding how the present study compares to Koch’s postulates as all other leaf nodule ‘obligate symbioses fail to satisfy them. Furthermore, the functional value(s), relayed to them being obligate, is still in question, as is the association in the present study.

We thank the reviewer for the thorough and constructive comments. We addressed all the comments made on the annotated PDF file in the revised version of the manuscript (see version with tracked changes).

We understand why the referee would like us to address the fulfillment of Koch’s postulates, but we would like to remain cautious. First, Koch’s postulates are traditionally applied to pathogenic interactions, or at least to interactions which lead to visible symptoms or signs. This can probably be turned around in case of a clearly mutualistic association with clear benefits or morphological changes, but so far we were unable to detect such features in the *Dioscorea/Orrella* symbiosis. Until we can unambiguously assign traits that are dependent on the presence of a symbiont, we would like to refrain from addressing these concepts to avoid potentially misleading some readers.

References: match those listed in text and are appropriate to this study, and useful to any researcher interested in this area of inquiry;
Table 1: clear results;
Table 2: clear results;
Fig. 1: see various comments made in the legend. Satisfying the comments should greatly improve the quality of the images;

We modified the legend as requested. One question posted on the PDF was whether Fig. 1E and 1F showed different sides of leaves. The images in fact show the adaxial face of the leaf in both cases. This is most apparent when looking at the tip of the leaf. Perhaps the somewhat different orientation of the lighting on both images gives the impression that different sides of the leaves. To avoid confusion, we specify in the revised legend that the adaxial side is shown.

Fig.: 2: see comment in legend;

The reviewer asks why the gland shows red fluorescence. We believe that the very high density of bacteria in the leaf gland is solely responsible for the fluorescence. The somewhat granular appearance is likely due to bacterial aggregates or microcolonies in a diffuse matrix of EPS or unidentified mucus.

Fig. 3: Clear results;
Fig. S1: detailed, clear, and ok. Images of good quality;
Fig. S2: detailed, clear, and ok
Fig. S3: detailed, clear, and ok
Table S1: detailed, clear, and ok
Table S2: detailed, clear, and ok
Table S3: detailed, clear, and ok

Thank you for your constructive comments and for your time reviewing this manuscript.

## Reviewer #2

Really nice work, you can see that even though it’s the most ‘easy’ interaction in the bacterial leaf symbiosis, it’s still a handful to work with these symbiotic plants, especially due to their longer life cycle and difficult plants to propagate.
So congratulations for your manual work and results.

Many thanks for the kind comments.

Main remarks

I have some remarks on the main text.
• There were a lot of beauty flaws in your work
o Check your reference style

♣ Some scientific names in the references are in italics, others are not.

We corrected mistakes in formatting references in the revised text.

o Use your units consequently
♣ if you use L, use than mL
♣ Use spaces between amount and unit
♣ Stick to which notation you want to use: 37°C, 37 °C, 20 g/L NaCl, 20g/l NaCl, NaCl 20 g/L or NaCl (20 g/L). All notations are well as long as you don’t see them all in the same paper (R144 vs R145). Be consistent with the use of capitals and space.

We corrected inconsistencies in notation and units throughout the manuscript, e.g. at L. 188, 192, 146-148.

o Use sub and superscript when you write chemical formula (R184, R 244)

We corrected formulas throughout the text, where appropriate (e.g. L189)

o When are amounts written out in full? Under 10 (not followed by a unit) or at the beginning of a sentence? Both rules are sometimes followed. Be consistent

We corrected several instances of such style mistakes throughout the manuscript.

o Be sure to explain your abbreviations in full before you use them.
♣ MS(R108), TSB (R122), EMS (R173),…

We made sure the abbreviations were defined at first mention: MS at L. 110, TSB at L 113 and EMS at L. 177.

♣ Are some abbreviations really necessary? PO (R173) , RT (R180) or MIC (307)

Indeed. We removed these abbreviations and instead spelled out the names at L.177, L312 and L183.

• Are all the adjustments of your material & methods written in your results present in your material and methods? There is a strange flow during the material & methods, while the story comes clear in the results. I would advise to structure the m&m more, perhaps the use of a set the scene in the start of M&M could help the reader to show how you worked. In the methods all the methods seem to be done all at once, while the results show it was a process of elimination to be able to make a aposymbiotic plant.

We reorganized the structure of the M&M section to improve the flow of the manuscript, by arranging the different sub-sections in a more logical order.

• There is no data on the development of first part of the results (Symbiotic D. Sansibarensis are recalcitrant to inoculation with exogenously applied O. dioscoreae). Is it possible to add some data in the supporting information?

Negative results are always tricky to present properly. We tried to give some more details about the results at L.289 and 297, for example.

• It is not clear which statistical tests were used to validate the results. Can you specify and add these tests and the p-values?

The tests performed and p-values are specified in the figure legends (e.g. legends of Figure S2, S3, and Figure 3).

• In the result section, references were added to support some methods, these references fit more in the method section than the result section.

We deleted citations to references 28, 52, 57 and 58 from the results section, and moved them to the methods section as suggested.

• In the discussion, it is not clear which method was the best option to develop an aposymbiotic plant. In addition, none of the methods is sound, there is always a possibility of infection. Do you have ideas to further investigate and search for a better method?

We apologize for this oversight. We added a short paragraph in the Discussion at L429-431 to remind the reader that in vitro antibiotic treatment of node cuttings followed by regeneration of whole plants is the most effective and reliable method to obtain aposymbiotic plants. As with any in vitro propagation method, there is always a possibility of contamination from endophytic microorganisms but most likely from exogenous sources. Rigorous sterile techniques and quality control of media, reagents and equipment are still the best way to control these contaminations.

1. Introduction

• R38 Be consistent. If you refer every time to a plant – bacteria interaction. It is maybe best to address the symbiosis as Medicago/Sinorhizobium. In the rest of your paper, you are talking of D.sansibarennsis/ O. dioscoreae.

We corrected this as suggested at L39.

• R69. Why is it important to state that nitrogen fixation is ruled out in the Dioscorea/Orella symbiosis, is it not in the other leaf nodulating interactions?

Older (pre-1970s) literature mentioned that putative leaf endophytes isolated from Psychotria species could fix nitrogen (see Silver, Centifanto and Nicholas 1964, for example). This finding was later invalidated by other authors, but unfortunately the idea that leaf nodule bacteria are responsible for nitrogen fixation remains pervasive in non-specialist literature. ChatGPT itself rather ambiguously mentions this as of January 2024, when asked about the function of leaf nodules in Psychotria:

“[…] Leaf nodules, as traditionally understood, are structures typically associated with nitrogen-fixing bacteria in the roots of certain plants, such as legumes.”

We thought it important to keep mentioning in our articles that there is no evidence of nitrogen fixation in leaf glands or nodules, if only to enable better training of the further generations of AI bots. In the context of this manuscript, it would perhaps seem like an oversight not to assess plant development of aposymbiotic plants under low-nitrogen conditions if it hadn’t been formally ruled out before.

2. Material & Methods
• R108: ½ MS vs R115 half strength Murashige and Skoog

We corrected at L119 for consistency.

• R116 16h/8h routine, maybe use this abbreviation for the rest of the methods (R132, R151, R244)

We changed to “16h/8h photoperiod” throughout the manuscript, where appropriate.

• R117: The injection in the bulbils was it at a specific location and depth or random?

This was done at random, as shoot growth centers are not easily detectable without dissection. We rephrased at L128 to “*O. dioscoreae* cell suspensions prepared as above were injected directly into surface-sterilized bulbils with a 26G needle at one randomly chosen site”.

• R124-R130: Strange construction, wouldn’t it be better to put the names of the different methods between brackets after each sentence?

We rephrased at L146-156 as suggested.

• R134 Could it be possible to state that you will use two surface sterilisation protocols? The bleach+ ethanol protocol and the PPM protocol

We added a sentence at L. 166 as suggested.

• R143: you write what is present in MS medium, is that the same in R108, R115, R140?

The composition given here is of MS medium with supplements, while earlier mentions are of MS basal medium. We slightly rephrased L176 and L110 to avoid confusion.

• R143 2% sucrose is that (w/v) like R215?

Indeed. We corrected this at L176.

• R153-R159: suggestion: would a table with all the Orella strains, with the specific characteristics not be a good addition?

Details on bacterial strains are given in Table S1. We apologize for omitting to cite the table in this section of the M&M. We corrected the manuscript accordingly at L. 113.

• R153-159: why did you use both tags GFP en mCherry?

This was mostly for practical reasons. GFP was easier for us for counting colonies and imaging on solid media because our lab is equipped with a dissecting scope with the appropriate filters. Bacteria tagged with mCherry are otherwise better for in planta imaging with confocal microscopy due to the lower autofluorescence levels in that channel.

• R196 a verb is missing. Is this the triple A-staining mentioned in Figure 1?

This was corrected at L. 214

• R236 ‘Leaf length, width, area, and acumen length were determined from photographs…’ was this used to calculate the leaf area

Leaf area was calculated from manually segmented images using standard functions available in the Fiji software suite.

• R240: which statistical analysis was used?
• R269: which statistical analysis were used?

We specify the test in the figure legends and at L. 250 and L. 281 of the revised manuscript.

3. Results
• If you are not combining results and discussion, it’s strange to see references in this part. (R275) Could these references be integrated in the discussion or the methods??

As indicated in response to an earlier comment, we restructured the manuscript to remove citations to methods from the Results section.

• Table 1: Maybe add in the caption which effect you were researching and the contact time.

We rephrased the title of Table 1 to improve clarity. Contact time for each experiment is indicated in a separate column in the table.

• R273-296: why were the amounts of bulbils and shoot tips not mentioned in the material and methods.

We thought that mentioning the number of replicates or data points directly in the Results section improves clarity, since all the results mentioned in the first section are “negative”, i.e. consistently yielded symbiotic plants and statistical analysis on such data is irrelevant. As such, we hope it is easier for the reader to get an idea of how representative these “negative” results are.

• R298-R328. This is a mix of Methods (some of the methods explained here are not mentioned in methods), results and discussion.

We carefully read this section, corresponding to the paragraph “Treatment of node cuttings with an antibiotic cocktail results in aposymbiotic plants”, which relates the results of antibiotic sensitivity assays on bacteria in vitro and in planta, as well as assays to detect off-target activity of the antibiotics on the explants. Aside from details of the experiments which we believe are essential to understand the results, we did not readily identify sections which clearly belonged better in M&M rather than the Results section. Perhaps the reviewer was referring to another section of the manuscript?

• R341 how did you test the presence of bacteria? Molecularly?

We tested for the presence of bacteria in leaf glands by serial plating as detailed in section “Detection and identification of bacteria” of the M&M.

• Table 1:
o maybe add contact time and which effect you were researching in your caption

See answer to previous comment.
o 10°, does that mean that 1 or none CFU were found?

This is a strange notation indeed. We changed <10^0^ to 0 in the table.

This
• Figure 1:
o why no white circle around G en H?

We corrected this oversight in the revised Figure 1.
o why is the resolution different between G-H, I-J, M-N?

We tried as much as possible to obtain representative images at similar scales, but this was unfortunately not always possible. We chose these specific microscopy images in Figure 1 because they showed a comparable level of details of homologous structures.

o Caption:
♣ what is triple A staining, not mentioned in the text

This is more or less lab jargon, and “triple A” refers to the Astra blue, acridine red and chrysoidine stain described in the materials and methods. We corrected the legend of figure 1 to make this clearer.

♣ C and D, the mucus, trichomes and bacteria can they be visualised?

♣ K and L, can the trichomes be visualised.

♣ L remove (L) ♣ M I would not abbreviate ER

The revised legend of figure 1 was rephrased as suggested and specifically to be less ambiguous, to: “Cross-section of *D. sansibarensis* gland stained with acridine red, chrysoidine and astra blue showing a dense, orange-colored mixture of mucus and bacteria filling the lumen of symbiotic glands”. Similarly, panels K and L show trichome cells rather than whole trichomes.

• Figure 2:
o Caption.
♣ Is it not possible to emphasise more that the gland is recolonised? ♣ mcherry? Is it necessary to put it in italics here cf. there results of the GFP-strain R277

We slightly rephrased the legend as per the reviewer’s suggestions, adding the sentence: “[…] **B**. Close-up of a *D. sansibarensis* leaf gland colonized by mCherry*-*tagged bacteria (R-71417).”

• R392-R407: you mention 24 plants, while in the methods, you start with 25 plants. In addition you mention in the methods that 18 plants were aposymbiotic, while in the results you only mention 14.

Thank you for catching this. We forgot to mention that 1 plant died soon after transfer from Microbox to open pots. In addition, 3 of the “aposymbiotic” plants went into growth arrest soon after inoculating and were censored from the final dataset since the reason for the growth arrest was unclear. We added the following to the M&M section at L258-260: “One aposymbiotic plant died at the beginning of the experiment, and 3 others went into growth arrest in the following days, possibly due to stress from the transfer from sterile containers to open pots.”

• R398 which method of inoculation could give a better option, this dripping option was also not a good option with the aposymbiotic bulbils?

The “dripping” method is highly effective (>95% success rate), as long as the plants are kept in sterile conditions before inoculating. We modified L.439-444 of the discussion to explicitly remind the reader of this.

• R404 which statistical test did you use to test whether the results were significant.

Because the sample distributions did not satisfy the formal requirements for ANOVA, we resorted to non-parametric Kruskal-Wallis tests, grouping observations by date. We now specify this in the revised M&M at L277 and in the legend of Figure 3.

• R405-R407: why do you refer first to B and C and then to A.

We rephrased this paragraph at L414-416 to correct this.

• Figure 3:
o Caption refers to stem lengths in C, while the graph refers to shoot length

We apologize for the oversight. We corrected the axis legend.

o Caption refers to the total leaf area of individual plants tracked over a period of 30 days. What are the dots and what is the line?

The dots are the actual data points for each plant over the experiment, colored by condition. The solid lines depict the moving average per condition. We amended the legend of figure 3 to add these details.

o C I would remove the abbreviations apo, control and sym if you work with a colour legend

We removed the abbreviations from the horizontal axis as per the reviewer’s suggestion.

o C are the dots around the boxplots the values? Why are these not on one line per phenotype? These dots are not identified in the caption or in the legend.

The dots are indeed the actual data points, with “jitter” added to make near overlapping values more visible. The somewhat larger dots represent outliers. We edited the figure legend to make this clearer.

4.Discussion
• R439 Not clear here, if leaf nodules in Psychotria develop when the bacteria is absent.

Given the difficulty of working with *Psychotria* species, and the fact that aposymbiotic *Psychotria* sp. often suffer other developmental defects (see Ref. 17 for example), we agree that the causal link between formation of leaf nodules and the presence of the bacterial symbiont is difficult to demonstrate unequivocally. We rephrased our statement for clarity at L. 452: “This is in contrast to leaf nodule symbiosis in the *Psychotria* genus, where aposymbiotic plants present few, abnormal or no leaf nodules”

• R450 Psychotria kirkii is a synonym of P. punctata

Thank you for pointing this out. We added this detail at L. 466.

5.References
• Use italics for scientific names
• R534: somethings wrong with the pages of this article

We corrected reference format and mistakes, where applicable.

6.Supporting information
• Figure S1:
o Add white circles to make the letters readable and consistent.
o S should be a small letter and the same font size as b. (maybe use arrows to visualise specific structures
o I can’t find s on the figure E

Figure S1 was modified according to the reviewer’s suggestions.

• Figure S2:
o Is it possible to use a bigger font size on the axis
o Why are the values spread over the X-axis, should be one line per condition (same remark as figure 3)
• Figure S3
o Is it possible to use a bigger font on the axes

We modified Figure S2 and S3 as per the reviewer’s recommendation. As previously, we added “jitter” on the dot representation of the observations to avoid obscuring overlapping data points

o Use the same layout as Figure S2

We paid attention to use the same colors and naming conventions between the 2 figures.

o Why are the values spread over the X-axis, should be one line per condition (same remark as figure 3)
• Table S1:
o why not mentioned in the methods (R152-159)

We now added a mention to Table S1 in the Materials and Methods.

o Maybe add in which experiment they were used?

Where each strain was used is mentioned in the main text (Results section) or in Table S2.
o Why was E.coli used?

E. coli was used as a host for some plasmids used in the generation of tagged strains, but is irrelevant for this manuscript. We removed it from Table S1 and made minor corrections to the table.

• The data underlying the statistics are missing, can these be added as well?

All raw data used throughout the paper are available from a public repository, as mentioned in the “Data availability” section: <https://doi.org/10.57745/R0VPGY>
